# Estimation of Dietary Exposure to Contaminants Transferred from the Packaging in Fatty Dry Foods Based on Cereals

**DOI:** 10.3390/foods9081038

**Published:** 2020-08-01

**Authors:** Antía Lestido-Cardama, Ana Rodríguez Bernaldo de Quirós, Juana Bustos, M. Luisa Lomo, Perfecto Paseiro Losada, Raquel Sendón

**Affiliations:** 1 Department of Analytical Chemistry, Nutrition and Food Science, Faculty of Pharmacy, University of Santiago de Compostela, 15782 Santiago de Compostela, Spain; ana.rodriguez.bernaldo@usc.es (A.R.B.d.Q.); perfecto.paseiro@usc.es (P.P.L.); raquel.sendon@usc.es (R.S.); 2 National Food Center, Spanish Agency of Food Safety and Nutrition, E-28220 Majadahonda, Spain; JBustos@mscbs.es (J.B.); MLomo@mscbs.es (M.L.L.)

**Keywords:** fatty cereal based foods, multilayer polymers, dietary exposure, screening, food contaminants, GC–MS

## Abstract

Food packaging has received special attention from the food safety standpoint since it could be a potential source of contamination through the migration of chemical substances from the packaging material into food. The assessment of the exposure through the diet to these contaminants from food packaging is necessary. In this work, an estimation of dietary exposure of the young Spanish population (1–17 years) to target chemicals from packaging for fatty dried foods based on cereals was assessed. For this purpose, a gas chromatography coupled to mass spectrometry (GC–MS) method was developed for screening of volatile and semivolatile compounds, potential migrants from the packaging. Then, this technique was used to quantify 8 target analytes, which were previously identified in the packaging (including phthalates, acetyl tributyl citrate (ATBC), butylated hydroxytoluene (BHT) and octocrylene), in composite food samples of fatty cereals prepared according to the consumption data for different age groups. Among the phthalates, exposure to diethyl phthalate (DEP) was the highest for the three groups considered (0.0761–0.545 µg/kg body weight/day), followed by bis(2-ethylhxyl)phathalate (DEHP), while the lowest mean intake was found for di-n-octyl phathalate (DNOP; 0.00463–0.0209 µg/kg body weight/day). The estimated dietary exposures did not exceed for any of the analytes the corresponding established tolerable daily intakes.

## 1. Introduction

Food packaging has become an indispensable tool in food manufacturing since it protects the food from contamination (chemical, biological and physical) retaining its nutritional properties and sensory characteristics, which extends the shelf-life of the product [1,2]. Polymeric films are commonly used as food packaging due to their versatility; they are easily processed and can acquire different shapes and sizes. In some cases, the functionality and properties of this material are further enhanced by combining different polymer layers to form a multilayer structure where each layer develops a specific function [2].

Despite the advantages that packaging provides to the consumer, there are many debates concerning environmental and health topics since food packaging could represent a potential source of contamination. Food packaging materials are not only composed of intentionally added substances (IAS; e.g., monomers, additives as antioxidants, lubricants, plasticizers, etc.), they could also contain the popularly known as non-intentionally added substances (NIAS) like decomposition products, reaction intermediates, impurities, etc., which could migrate from the packaging into food, particularly into fatty foods [1,3]. This fact is undesirable, but a certain transfer is inevitable because currently the majority of foodstuffs are commercially packaged. The problem arises when the quantities of these migrating compounds that are transferred into foodstuffs may endanger health of the consumer or cause unacceptable changes in the composition or organoleptic characteristics of the food [4].

Due to the growing consumer awareness in terms of health issues, the migration process of chemical compounds from food contact materials (FCM) to food attracted the attention of the legislative communities. The EU and the Food and Drug Administration (FDA) began a global control to ensure consumer safety with the creation of positive lists of substances authorized to be used in the manufacturing of packaging material with their specifications, while restricting chemical substances with toxic potential [1]. Regulation (EU) No 10/2011 sets restrictions and specifications on the use of certain substances to determine the compliance of plastic materials and articles intended to come into contact with food [5]. However, other materials such as paper, glass, metals or components used in combination with plastic materials like printing inks, adhesives and so on are not regulated yet. For all these substances it is necessary a risk assessment with the final objective of ensuring that they do not represent a risk for human health [4].

An important part of risk assessment is the estimation of the dietary exposure to chemical contaminants from the FCM. One of the methods used to investigate population exposure to chemical contaminants through the diet is the so-called total diet study (TDS), which turns out to be a convenient and economical approach. The main characteristics in this type of study are that it should include a selection of foods representative of the total diet, the food is analyzed as consumed and grouped into pools resulting in more realistic exposure estimation [6]. Nevertheless, TDS is not able to show what type of food product is the principal contributor to contaminant exposure [7].

Our study is based on a TDS-like investigation because it does not cover the total diet, but focuses on specific food groups, concretely fatty dry foods based on cereals [8]. Our group has previously published exposure data on cereal-based foods, but of a “non-fatty” nature [9]. It is known that the fat content of food products is a factor that affects the migration process. For many chemicals, for example plasticizers, the amount that migrates is greater in fatty foods compared to low-fat content foods. This increase commonly is due to the higher solubility of the organic migrants in fat [10,11].

A total of seven samples including snacks and biscuits packaged with plastic materials were selected to be analyzed. In a first step of this work, a screening approach was applied to simply and rapidly determine the identity of potential chemical migrants in the packaging through the solvent extraction technique, followed by gas chromatography coupled to mass spectrometry (GC–MS). This is the most widely applied technique for identification of unknown compounds due to its reproducibility, robustness and the availability of standardized commercial libraries [12]. The type of packaging material was identified by IR with attenuated total reflection (ATR).

The aim of the second part of the study was to develop a method for the quantification of potential contaminants, previously identified in the packaging materials, in the real packaged foods. This migration assessment is necessary but at the same time challenging due to the complexity of the food matrix. For that purpose, samples were grouped into different pools according to the consumption data [13] of different age group (1–2 years, 3–9 years and 10–17 years) and homogenized to be extracted. The analysis of the extracts was performed using the GC–MS technique and data were acquired in the selected ion monitoring (SIM) mode.

The final objective of this work was estimating the dietary exposure to certain chemicals transferred from FCM by combining, data on the obtained concentration of the selected chemical contaminants in the pool samples, with national data on their consumption. Child and adolescence population (1–17 years) was selected because they may be considered as a potentially vulnerable subgroup to the toxic effects of chemical contaminants in food due to their organs are still under development during this age. Furthermore, child and adolescence population consume a greater amount of drinks and food compared to adult population (expressed per kg of body weight), which leads to reasonably higher exposures to chemicals with possible harmful effects [14].

## 2. Materials and Methods

### 2.1. Reagents and Standards

Acetonitrile for liquid chromatography (ACN), ethanol absolute for analysis (EtOH), methanol for gas chromatography (MeOH) and n-hexane for gas chromatography ECD and FID (HEX) were provided from Merck (Darmstadt, Germany).

Analytical standards with high purity were used in the study. Butylated hydroxytoluene (BHT) 99%, di-n-octyl phthalate (DNOP) 99.5%, bis(2-ethylhexyl)phthalate (DEHP) 99%, acetyl tributyl citrate (ATBC) 99% and as an internal standard diethyl phthalate-3,4,5,6-d4 (DEP-d) 99.3% were purchased from Fluka (Steinheim, Germany). While diethyl phthalate (DEP) 99.5%, dibutyl phthalate (DBP) 99%, diisobutyl phthalate (DIBP) 99% and octocrylene (OCTO) 97% were provided by Sigma-Aldrich (Schnelldorf, Germany). The chemical structures and the physicochemical characteristics of the analytes studied are given in Table 1.

Other analytical standards were used for identification: 4,4′-diphenylmethane diisocyanate and toluene-2,4-diisocyanate were obtained from Merck (Darmstadt, Germany). Triacetin ≥ 99% was purchased from Fluka (Steinheim, Germany). Toluene-2,6-diisocyanate 97%, 2,6-di-tert-butyl-1,4-benzoquinone 98%, methyl palmitate 97%, glyceryl trioctanoate ≥ 99%, benzophenone 99%, antioxidant 425, 1,3-docosenamide > 85%, 2,4-di-tert-butylphenol 99%, caprolactam > 99%, squalene ≥ 98% and saturated alkane standard mixture (C7-C30) were purchased from Sigma-Aldrich (Schnelldorf, Germany). Hexadecanamide 95% were provided by Combi-Blocks (San Diego, CA, USA).

For quantification purposes standard solutions at a concentration of 1000 mg/L were individually prepared in methanol for DNOP, OCTO, DEHP, DBP, DIBP and DEP and in ethanol for ATBC and BHT. An intermediate mix solution with a concentration of 10 mg/L was prepared in methanol. Calibration solutions in methanol were prepared by serial dilution from this intermediate solution and all of them contained the internal standard DEP-d prepared in ACN at a concentration of 0.5 mg/L. These solutions were stored at 4 °C in brown glass bottles and brought to room temperature before each analysis.

Several preventive measures were taken to reduce the contamination related with the ubiquity of phthalates (overestimations or false-positive results). Nitrile plastic gloves were used during all analytical work, sample manipulation was done avoiding the use of plastic materials, and the laboratory glassware was washed with an organic solvent and kept in the muffle oven at 400 °C (EML, Carbolite Furnances, England) during 4 h and then was covered with aluminum foil until its use.

### 2.2. Samples

A total of seven food-packaging samples of dried-fatty foods comprising snacks and biscuits based on cereals, all packed in plastic material and with different brand names, were purchased from a supermarket of Spain and taken to the laboratory for screening analyses. Detailed information was recorded from the labeling of each food, including data like carbohydrate/sugar, protein content and fat/saturated fat. The samples included in this study were selected since they are consumed widely by the young Spanish population in accordance to the national dietary survey ENALIA [13]. Each packaged food was sampled in duplicate and was stored frozen at −30 °C until analysis.

Samples were divided into three groups: snacks (AS; *n* = 4), popcorn (PL; *n* = 1) and biscuits (GA; *n* = 2). Information of the samples selected is shown in Table 2. The fat content of the samples ranged from 10% to 50%.

An Electronic Digital Outside Micrometer (Mitutoyo, Japan)) was used for thickness measurements of the packaging materials. The reported value was an average of three measurements.

### 2.3. Samples Preparation—Packaging Materials (Screening)

Two different extraction conditions were evaluated. An extract of the packaging, with a certain surface area of 0.8 dm^2^, was obtained by immersion of ten pieces of approximately 1 cm × 8 cm in 25 mL of ACN solvent and stored at 70 °C in an oven during 24 h. While another extract was obtained by immersion in 25 mL of hexane at 60 °C for 4 h.

For the GC–MS analysis, 10 mL of the obtained extracts were evaporated using a stream of nitrogen operated at 40 °C (RapidVap Vertex Evaporator, Labconco, Kansas City, MO, USA) up to one milliliter and the extract was filtered using a PTFE membrane filter of 0.45 µm (Membrane Solutions, Auburn, WA, USA) and injected into the chromatograph.

### 2.4. Sample Preparation—Foodstuffs

Some of the chemicals that were identified in the food packaging were chosen for later analysis in real foodstuffs with the objective to determine the exposure to them.

Individual samples were homogenized with a grinder, weighted and composite samples (pools) were elaborated taking into account the national consumption data corresponding to three groups of age (1–2, 3–9 and 10–17 years), and stored at 4 °C pending analysis. Each pool sample was prepared in duplicate (*n* = 2). For extraction, aliquots of one gram of the pooled sample were weighted and standard solutions in methanol were added at different concentration levels, 0.05, 0.1, 0.25 and 0.5 µg/g for the standard addition method (stand for 15 min to infuse into the foodstuff). The sample was extracted with 10 mL of ACN and stirred for 2 min with vortex agitation (VELP scientifica vortex). Then, the mixture was centrifuged at 1357× *g* for 10 min at −5 °C and the supernatant was collected and evaporated until dryness using a stream of nitrogen at 40 °C (RapidVap Vertex Evaporator, Labconco). The residue was reconstituted in one milliliter of methanol containing the internal standard DEP-d at a concentration of 0.5 mg/L. The extract was further filtered through a 0.45 µm PTFE membrane filter (Membrane Solution, China) and injected into the GC–MS. Each pool sample not spiked was analyzed in duplicate. Additionally, in each batch of samples a procedural blank was included to control the background contamination. Quantification of the analytes in the pooled samples was performed using the calibration curve obtained by the standard addition method, by plotting the peak area/internal standard ratio versus the added amount of each standard. In this way, the matrix effect was prevented in the quantitation.

### 2.5. Exposure Estimation

Dietary exposure was estimated taking into account the measured concentration of the selected analytes in each pool sample and the national consumption data for this type of food (ENALIA, 2014). According to GEMS/Food-EURO recommendations, for analytical results between limit of detection (LOD) and limit of quantification (LOQ; non-quantifiable), values were set to LOQ/2 to estimate dietary exposure [15].

ENALIA is a dietary survey conducted in Spain, between November 2012 and July 2014, in order to collect food consumption data. The methodology in this survey followed the European Food Safety Authority (EFSA) EU Menu guidance recommendations. This study included 1780 individuals with ages ranging from 6 months to 17 years. However, the age range between six and eleven months was ruled out due to the low consumption of the foods included in this study.

A risk assessment associated with dietary exposure was evaluated comparing the obtained chemical intake values with the available tolerable daily intake (TDI) values set by authorities such as EFSA and the World Health Organization (WHO).

### 2.6. ATR-FTIR

To identify the type of packaging material, an ATR (attenuated total reflectance)-FTIR (Fourier transform infrared) spectrometer (ATR-PRO ONE, FTIR 4700, Jasco, Tokyo, Japan) fitted with a diamond optical crystal and controlled by the Spectra ManagerTM v.2 software was used. Before the analysis, the samples were cut, cleaned with an organic solvent and dried. Infrared spectra were acquired on internal and external material surface and in the region from 4000 to 650 cm^−1^. The spectra identification was carried out using the KnowItAll 17.4.135.B software by comparing the sample spectra with the available infrared spectral libraries of polymers and related compounds from Bio-Rad Laboratories, Inc. (Philadelphia, PA, USA ). Results are shown in Table 2. 

### 2.7. GC–MS Method

GC–MS instrument was a Thermo Scientific Trace 1300 Series GC with a simple quadrupole Trace ISQ LT mass detector under electron impact ionization mode (EI) and an automatic injector AI 1310 (Thermo Fisher Scientific, San José, CA, USA). The ZB-5MS 5% phenyl and 95% dimethylpolysiloxane column (30 m × 0.25 mm × 0.25 µm) purchased by Phenomenex (Torrance, CA, USA) was used.

For screening purposes, 1.0 µL of the extract was injected in the splitless mode and the injector temperature was set at 300 °C. The column was set at a constant flow rate of 1 mL/min using helium (3X quality, from Nippon Gases) as carrier gas. The initial temperature of the oven was maintained at 40 °C for 2 min, then increased at a rate of 9 °C/min until reaching 300 °C, with a holding time of 3 or 10 min depending on whether the extract was acetonitrile or hexane, respectively. Electron impact ionization (EI) was set at a voltage of 70 eV. The temperature of the transfer line and detector was set to 300 °C. Data acquisition was operated in full scan mode with an m/z range of 30–500. Calibration of the mass spectrometer was performed by the autotuning mode in ISQ Dashboard software using the masses 69 and 219 of perfluorotributylamine (PFTBA). Xcalibur 3.0.63.3 software (Thermo Fisher Scientific Inc., San José, CA, USA) was used for data acquisition and processing. The tentative identification of compounds was performed using the libraries Wiley Registry^™^ 8th edition with 399.383 mass spectra and the NIST/EPA/NIH 11 v.2.0 containing 30.898 mass spectra.

For quantification purposes, injections were made in the split mode (ratio 1:5), the oven gradient used was initially at 60 °C for 2 min, then increased until 300 °C at 10 °C/min, with a holding time of 2 min, and data were acquired in the SIM mode using the quantification and qualifier ions presented in Section 3.2.

To minimize the potential source of phthalates contamination, several cycles of washing the syringe with organic solvents before and after injection were performed, and the washing solvents were changed every day.

## 3. Results and Discussion

### 3.1. Screening of Chemical Migrants in Packaging Materials

**Figure 1:**
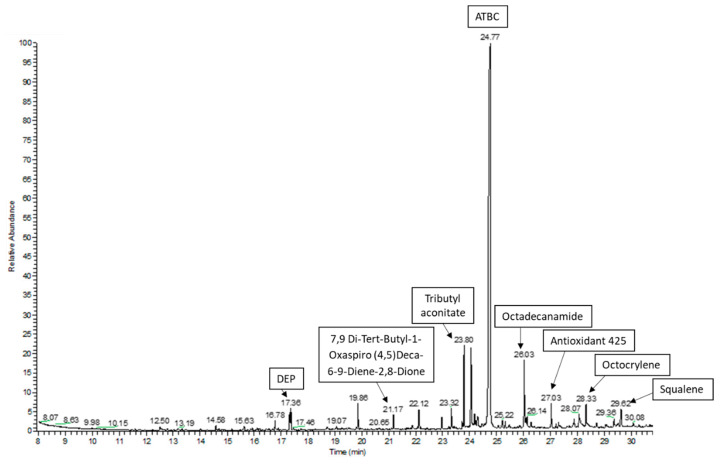
GC–MS chromatogram of the acetonitrile extraction in the sample GA_02.

In this study, a GC–MS screening analysis was used to identify potential chemical migrants that could be present in the studied plastic packaging materials. All detected peaks with the best matches found during the library search were considered in the study. The followed approach allowed one to tentatively identify or confirm more than 60 compounds of different nature in the packaging materials (Table 3). Figure 1 shows the GC–MS chromatogram for the acetonitrile extraction in the sample GA_02.

Some compounds could be confirmed with the injection, under the same conditions, of the available standard comparing the retention time and the corresponding spectral data; thus, close to half (28 compounds) could be positively confirmed. The remaining detected peaks were tentatively identified as it has been detailed in Section 2.7. Only compounds with the best matches (≥800) were considered in the study.

The Cramer decision tree allows one to estimate the toxicological hazard of a compound according to its molecular structure. For this purpose, the Toxtree v3.1.0 (Ideaconsult Ltd., Sofia, Bulgaria) software was used. The model classifies the molecules into three classes: class I (low toxicity), class II (intermediate toxicity) or class III (high toxicity) [16]. The threshold for Cramer Classes I–III are 1800, 540 and 90 µg/person/day, respectively [6].

Several substances related with the manufacture of plastic packaging materials like phthalates and other additives such as plasticizers, antioxidants, slip agents, ultraviolet (UV) filters or photoinitiators among others were identified.

Phthalates esters (PAE) are synthetic organic chemicals introduced in the 1920s [17]. This is a wide group of chemicals used in the manufacture of packaging materials, because they are used as plasticizers to impart flexibility and durability of polymeric matrices. However, numerous studies identified them as endocrine-disrupting chemicals and an important source for human exposure to phthalates seems to be the packed food, especially fatty-food are the most affected [5,17,18].

Detected low molecular weight PAE, such as DEP and DBP, are most frequently used in lacquers, adhesives, printing inks, varnishes and coatings. DIBP is a plasticizer for nitrocellulose, cellulose ether and polyacrylate and polyacetate dispersions [6]. Whereas, identified longer/branching alkyl chain phthalates, like DEHP and DNOP, due to their economic convenience, are commonly used as plasticizers in the polymer and polyvinyl chloride (PVC) industry to improve workability, flexibility and handling properties [18]. DEHP is the predominantly used plasticizer in several applications with the approximately 51% of the global production [6].

All these phthalates were found in all samples and in both solvents, except DNOP, which was only found in one sample (AS_01). DEP, DIBP and DNOP are not included in the Regulation 10/2011, while DBP and DEHP are authorized with restrictions as additives in plastic food contact material with a specific migration limit (SML) of 0.3 mg/kg and 1.5 mg/kg, respectively [5]. DIBP, DBP and DEHP are listed between the ten phthalates diesters that have been classified as carcinogenic, mutagenic or toxic for reproduction substances, with the mention “Reprotoxic 1B” by the European Chemical Agency (ECHA) [19].

Adverse effects such as asthma and allergic symptoms have been associated to the exposure to environmental phthalate esters [18]. Phthalate metabolites have also been detected in breast milk, serum and urine [20].

The growing concerns about the toxicity of phthalates have led to search other plasticizers. Some alternatives of PAE were detected in the packaging materials studied such as esters of bioderived citric acid (triethyl citrate, acetyl triethyl citrate, tributyl citrate and ATBC), which are mainly used as plasticizers in polymers such as PVC and cellulose acetate; phosphate esters (tributyl phosphate), which are used as a flame retardant and as a plasticizer in the manufacture of nitrocellulose, plastics, and vinyl resins intended to be in contact with food; sebacates (dibutyl sebacate), which exhibit good compatibility with PVC matrix and are principally used as plasticizers in film coatings [6,21]; and aconitate esters (tributyl aconitate), which protect PVC against negative effects of light and heat [21,22].

Isopropyl myristate is another plasticizer used for cellulosic, pigment dispersant and binder, which was detected in one sample (AS_01) [21]. Triacetin, also known as glycerol triacetate, was identified in three samples of snacks (AS_01, AS_02 and AS_04). This compound, besides its use as a food additive, is a plasticizer used in cellulose and adhesives since it is able to provide flexibility and/or elongation allowing their deformation [23,24]. Diethylene glycol dibenzoate, which was found in the extract of ACN of two samples (AS_02 and GA_02), is a plasticizer for polyvinyl chloride acetate and component used in adhesives [25,26].

Benzophenone (BP), a substance commonly used as photoinitiator in UV cured inks, adhesives, coatings, varnishes and other materials, and also as a UV blocker to prevent photodegradation of the packaging polymers or its contents, was detected in 4 samples (AS_02, AS_03, PL_01 and GA_02). This compound is included in the positive lists of the Regulation 10/2011with a SML of 0.6 mg/kg [4,5,6,27].

UV filter compounds like 2-ethylhexyl salicylate, octyl methoxy cinnamate and octocrylene, whose main function is absorbing the ultraviolet light to protect the product, were identified in several samples [21,27,28,29].

The synthetic phenolic compound, butylated hydroxytoluene (BHT), has been detected in two samples (AS_02 and AS_04). BHT is used as a food additive and as a common antioxidant compound and stabilizer in polymeric materials. This small molecule with a tendency to migrate rapidly presents a SML of 3 mg/kg [21,23].

2,2’-Methylenebis(6-t-butyl-4-ethylphenol) also so-called Antioxidant 425 is another phenolic antioxidant with medium molecular weight commonly used in polyolefins packages detected in five samples (AS_02, AS_03, AS_04, PL_01 and GA_02) [30,31].

Among the substances identified in the packaging samples, there are some degradant products of antioxidants and they could be considered like non-intentionally added substances (NIAS). This is the case of degradation products of antioxidants such as Irgafos 168 and Irganox 1010, 3,5-di-tert-butyl-4-hydroxybenzaldehyde was also found in some samples, this compound has been described in the literature as a possible product of the metabolism of BHT), and 2,6-di-tert-butyl-4-nitrophenol, which is formed when 2,6-di-tert-butylphenol is nitrated [6,21,32,33,34,35].

Squalene, which was detected in all packaging material samples, is an ethylenic-unsaturated hydrocarbon used as an oxygen-scavenging agent in the material to extend the shelf life of compounds that are sensitive to oxygen [36,37].

Diisocyanate compounds were detected in several of the samples analyzed. Among other uses, they are especially employed as an intermediate in the production of polyurethane products and all of them are classified as class III according to Cramer rules [21].

As the extraction was carried out taking into account both sides of the packaging, some substances identified may come from the solvents, dyestuffs and pigments employees for the external printing ink such as 2,6-diisopropylnaphthanlene (DIPN) identified in five samples (AS_02, AS_04, PL_01, GA_01 and GA_02) [23,38], 2-naphthenol found in the ACN extract of the sample GA_02 [39], dehydroabietic acid identified in the ACN extract of four samples (AS_01, PL_01, GA_01 and GA_02) [27], caprolactam detected in one sample (GA_02) or isophorone identified in the ACN extract of two samples of snacks (AS_01 and AS_02) [4]. Other substances identified could come to the adhesives employees such as the dehydroabietic acid, methyl ester, found in three samples of snacks (AS_01, AS_02 and AS_04), which is a product of the thermal degradation/dehydrogenation of abietic acid, a tackifier used in adhesives [24,40]; or cis-11-eicosenamide, identified in two samples (AS_01 and GA_01), which is used as an adhesive and a component of coatings [21].

Several slip agents based on fatty acid amides currently used were identified. 13-docosenamide was present in all the samples analyzed, while octadecanamide and hexadecanamide were only identified in three samples (AS_02, AS_04 and GA_02). They are able to reduce friction resistance and all of them belong to Class III according to Cramer rules [21,36,41,42].

This study shows that there are a lot of compounds that are not included in the positive list of the Commission Regulation (EU) No. 10/2011, only 20 compounds of the total of 65 listed in the Table 3 are included in this regulation [5].

### 3.2. Method Validation

Among all the identified compounds in the packaging materials analyzed, eight target analytes were selected for its later determination in food samples and thus making it possible to carry out the exposure assessment, including five phthalates (DEP, DIBP, DBP, DEHP and DNOP), the plasticizer ATBC, the antioxidant BHT and the UV filter octocrylene. The selection of these compounds was based on the increasing concern to human health and/or their abundance in the packaging samples.

The analytical performance of the developed GC–MS method for the selected analytes, such as linearity, sensitivity and precision were evaluated, and the data is shown in Table 4.

Linearity was examined using standard solutions of a known concentration (mixtures of all analytes) prepared in methanol. The calibration range consisted of at least seven points spread from 0.0025 to 0.025 µg/mL depending on the substance, to 2 µg/mL, the highest concentration tested. An internal standard (DEP-d) was added in order to compensate instrumental variability. Each calibration point was injected by triplicate. The relationship between known concentrations and measured area for each quantification ion relative to the area of the internal standard ratio (ion 153) was assessed by linear regression. The coefficients of determination obtained in the study indicated very good linearity with values equal or greater than 0.9930, so the method proved to be appropriate for quantification of these compounds in this concentration range.

The sensitivity was evaluated based on limits of detection (LODs) and quantification (LOQs). The quantification and detection limits were estimated as the lowest concentration, which provided a signal-to-noise higher than ten (the ratio between the peak area of quantification ion for each target analyte and peak area of noise) or three (the ratio between the peak area of qualification ion for each target analyte and peak area of noise), respectively. The method shows good sensitivity with LODs equal or lower than 0.01 µg/mL, corresponding to 0.01 µg/g of sample while LOQ obtained was equal or lower than 0.025 µg/g of sample. The precision, expressed as relative standard deviations (RSDs %), was determined in terms of repeatability analysis. Independent solutions in methanol at the level of 0.1 µg/mL were evaluated at different times on the same day (*n* = 8). The values obtained for repeatability (RSDr < 10%) were satisfactory.

The extraction method developed was examined in terms of recovery percentage. A way to study the recovery using the standard addition method is comparing the slopes of the standard addition line with the external calibration line in methanol. Each pool sample was spiked at four different concentrations (0.05, 0.1, 0.25 and 0.5 µg/g) the recoveries were in the range of 72–118%, as shown in Table 5.

### 3.3. Food Concentration of Selected Packaging Chemicals

Based on previous studies carried out in our laboratory, ACN was selected as an extraction solvent. This extraction solvent was also used for this type of chemical in cereal samples by other studies [3,9,43]. The second extraction step was considered unnecessary because the concentration determined resulted to be below 10% of the concentration determined for the first extraction.

Despite the efforts to reduce the background levels of contamination with phthalates, some of them were still found in the blanks, so an approach of subtracting the blank value from the concentrations measured in food samples was applied [7].

The quantification of the target analytes in the pooled samples was performed using the calibration curve obtained by the standard addition method. The concentration of selected migrants obtained in composite food samples (mean of two replicates) is shown in Table 5.

All of the analytes analyzed were found in the food samples, except DNOP, which was present at concentrations below LOQ in all composite food samples analyzed. This phthalate neither was detected in the food group of grains/cereals analyzed by Schecter et al. (2013) and Bradley et al. (2013) [7,44]. Regarding the other phthalates and octocrylene, the pool representing the 10–17 years age group consumption was the one with the highest values; except for the DEP for which the highest value, 0.329 µg/g was found in the pool of the 1–2 years group. Regarding ATBC and BHT, the highest values were found for the 3–9 years age group, 0.0554 µg/g and 0.0476 µg/g, respectively.

Overall, among the analyzed substances, the greatest concentration was obtained for the phthalate DEHP in the pool representing the 10–17 years consumers group (0.524 µg/g). This is in line with published observations that DEHP would be the predominant compound, in terms of concentration and frequency, in food, and this is related to its affinity for the fatty fraction [43,45,46].

Comparing these results with the previous study published by our group [9] focused on low-fat cereal based, it can be seen that the concentration of phthalates and octocrylene is higher in samples with a higher fat content, while the concentration of ATBC is lower, so there is not a clear trend between the concentration of ATBC and the fat content of the foods. Cao et al. (2013) developed a sensitive and selective GC–MS method for the determination of DEHA and several phthalates in food samples from the 2013 Canadian TDS, generating recent data for dietary exposure assessment [3]. DEHA and some phthalates, including DIBP, DBP and DEHP, were detected in cereal product samples, but at low levels in general. Reported levels of DIBP were in the range of 2.89–15 ng/g, for DBP 7.09–36.5 ng/g and for DEHP 18.8–153 ng/g. Our estimations are in the range of the reported DEHP and DBP values, except in the pool of the 10–17 years group, where we found higher concentration values (0.524 µg/g and 0.0426 µg/g, respectively). In the case of DIBP, our study found higher concentration values in the three tested pools (0.0344–0.102 µg/g). However, if we take into account the sample of popcorn included within the miscellaneous group in the study of Cao et al. (2013) [3], the range of values reported is extended to 0.284 ng/g for DEHP, 0.0398 ng/g for DIBP and 0.208 ng/g for DBP.

In another study Cariou et al. (2016) [43] developed a GC–MS/MS method to monitor the phthalates DIBP, DBP, BBP and DEHP in typical foodstuffs of the French diet. Among the cereal’s samples included in the study, it is worth highlighting the high concentrations found in the popped corn with bacon flavor samples: 40.3 ng/g for DIBP, 54.2 ng/g for DBP and 114 ng/g for DEHP. These values were higher than those obtained in the present study, except for DEHP in the pool of 10–17 years group and for DIBP in the pools of 1–2 years and 10–17 years.

More recently, Yang et al. (2018) [47] assessed the dietary exposure to 16 phthalate esters (PAEs) in the Chinese population by a TDS. Among the PAEs analyzed, three phthalates, including DNOP were not detected in any of the food samples just like in our study. Reported levels of DIBP, DBP and DEHP within the cereal food group were higher than our estimations, except for DEHP in the pool of 10–17 years. For DEP, our results showed higher concentration values in the three pools (0.205–0.3299 µg/g). The same occurs for these phthalates when we compare our results with the reported concentration values obtained for the group of snacks (salty biscuits, popcorn, etc.) found by Van Holderbeke et al. (2014) [46] and the group of miscellaneous cereal products in the study of Bradley et al. (2013) [44]. Except that DNOP is detected in the study of Van Holderbeke et al. (2014) [46] and that our reported level of DIBP in the pool of 10–17 years (0.102 µg/g) exceeds the higher level detected (0.083 µg/g) in the study of Bradley et al. (2013) [44].

Sakhi et al. (2014) [48] determined the concentration of several phthalates in Norwegian food and beverages. If we compare our results with those obtained in the food category of snacks (all of them packed in plastic material), the concentration of phthalates found in this work were higher.

### 3.4. Estimation of the Exposure to Selected Migrants and Risk Assessment

Dietary exposure of consumers to chemicals is a crucial element in risk assessment. Estimated dietary exposure values (mean and 95th percentile) to the selected migrants in the different age groups of the Spanish child and adolescent population are presented in Table 5.

Among the phthalates, exposure to DEP was the highest for the three groups considered, followed by DEHP, while the lowest mean intakes were found for DNOP. This result coincides with the reported fact that the food group that contributes most to the total dietary exposure to DEP is grains and grain-based products [45]. On the other hand, Sui et al. (2014) [49] showed that for children and adult groups, cereals resulted to be the food category that represents the top contributor to the dietary intake of DEHP.

In agreement with the estimation of dietary exposure to phthalates carried out by Fierens et al. (2014) in the Belgian population, where predicted dietary exposure rates for DEP and DBP decreased with the population age [45], the same observation can be inferred from the results in our study. The average dietary exposure to DEP ranged from 0.0761 µg/kg body weight per day in the pool of 10-17 years to 0.545 µg/kg body weight per day in the pool of 1–2 years. The 95th percentile exposure to DEP ranged from 0.397 µg/kg body weight per day in the pool of 10–17 years to 2.80 µg/kg body weight per day in the pool of 1–2 years. Regarding DBP, the mean exposure ranged from 0.0159 µg/kg body weight per day in the pool of 10–17 years to 0.0618 µg/kg body weight per day in the pool of 1–2 years, while the 95th percentile varied from 0.0831 µg/kg body weight per day in the pool of 10–17 years to 0.315 µg/kg body weight per day in the pool of 1–2 years. It is interesting to highlight that although the mean concentration of these analytes in the pool of 1–2 years group was not particularly high relative to the pools for other age groups, it became the higher contributor to the dietary exposure due possibly to the fact that this population group has the highest consumption of this type of products on a body weight bases [49]. Average daily consumption of Spanish population for selected products (snacks and biscuits) is 1.671 g/day (P95 8.508 g/day) for the 1–2 years group, 0.9806 g/day (P95 4.265) for 3–9 years and 0.3705 g/day (P95 1.932 g/day) for the 10–17 years group.

In contrast to the results for the above-mentioned phthalates, estimated dietary exposure levels to DEHP were higher in the adolescent group (10–17 years) than in the others. The mean exposure varied from 0.0995 µg/kg body weight per day (pool 3–9 years) to 0.194 µg/kg body weight per day (pool 10–17 years), while the 95th percentile varied from 0.433 µg/kg body weight per day (pool 3–9 years) to 1.01 µg/kg body weight per day (pool 10–17 years). However, the opposite happens in the estimation of dietary exposure to DEHP in the Chinese population carried by Sui et al. (2014) [49] where the mean dietary exposure decreases with age varying from 2.03 µg/kg body weight per day in the adult group (more than 18 years) to 4.51 µg/kg body weight per day in the children group (2–6 years). These estimated dietary exposure values to DEHP were greatest than those found in our work.

Schecter et al. (2013) [7] estimated the dietary phthalate intake for children and adults in U. S. foods obtaining a mean of 0.028 µg/kg body weight per day for DEP, 0.008 µg/kg body weight per day for DIBP, 0.035 µg/kg body weight per day for DBP, 0.136 µg/kg body weight per day for DEHP and 0.001 µg/kg body weight per day for DNOP in the group of grain where cereals and cookies were included. These findings are comparable with our values in the case of DEHP and DBP, except in the pool of 1–2 years group where we estimated a value two times higher for DBP (0.0618 µg/kg body weight per day). However, the estimated values in our study for DEP, DIBP and DNOP turned out to be higher than those reported in the work of Schecter et al. (2013) [7].

The comparison of our estimated dietary exposure with other studies previously reported in the literature should be interpreted with caution. Large variations may be due to several factors such as the type of foods included (our study was limited to fatty dried foods including snacks based on cereals and biscuits), the analytical method used to analyze the samples, the group of population selected (our study was limited to 1–17 years), the type of container for the food (our study was limited to plastic packaging), the different sources of exposition (our study was limited to food), variability in the consumption of foods according to the country, special dietary habits (vegetarians, vegan, etc.), etc. Even the occurrences of different compounds can also change over time, for example, the restricted phthalates like DEHP are gradually being replaced by others like DINP and DIDP [48].

For certain analytes, authorities and other organizations such as EFSA and WHO, have established TDI in order to protect the human health. This TDI is an estimation of the maximum daily exposure to a certain agent that the population may be exposed to without any large risk. In relation to our analytes, the TDI specified for DEP is 0.5 mg/kg body weight per day, 0.01 mg/kg body weight per day for DBP and 0.05 mg/kg body weight per day for DEHP [50,51,52]. No reference doses (RfDs) or TDIs have been set for the other analytes. Looking at our data, and taking into account these individual TDI, in all cases, the predicted dietary intake rates were far below the TDI values established. Even our highest mean dietary exposure value of 0.545 µg/kg body weight per day for the DEP in the pool 1–2 years turned out to be 1000 times less than its corresponding TDI.

Other substances identified in packaging materials, specifically BHT, ATBC and octocrylene were also considered in this study. For ATBC the mean dietary exposure varied from 0.0196 (pool 10–17 years) to 0.0652 µg/kg body weight per day (pool 1–2 years). Regarding BHT, the mean exposure varied from 0.00282 (pool 10–17 years) to 0.0471 µg/kg body weight per day (pool 3–9 years). The estimated dietary exposure to octocrylene ranged from 0.0119 (pool 10–17 years) to 0.0435 µg/kg body weight per day (pool 1–2 years). Ibarra et al. (2019) [9] estimated the dietary exposure to ATBC using low fatty cereals obtaining a mean of 1.01 µg/kg body weight per day in the pool 1–2 years, 2.01 µg/kg body weight per day in the pool 3–9 years and 1.27 µg/kg body weight per day in the pool 10–17 years. These results are higher values compared to those obtained in this work. In the case of ATBC, a comparation was made with the results obtained in the FACET exposure tool (Facet 3.0.2, 2008–2012 Creme Software Ltd., Dublin, Ireland) developed within the European Commission 7th Framework project. This software allows one to evaluate the exposure to some flavors, additives and chemicals from FCM through a probabilistic approach. In this case, consumption data from the UK survey were used and higher mean exposure values were found being 0.24 µg/kg body weight per day in the pool 1–2 years, 0.16 µg/kg body weight per day in the pool 3–9 years and 0.78 µg/kg body weight per day in the pool 10–17 years. It is important to highlight that there is little information in the literature related to the exposure to these analytes (octocrylene and BHT) from packaging material.

Briefly, the packed based cereal foods are an important dietary source of exposure to these chemicals, for example in the case of 1–2 years age group low and high fat content foods contributes similarly to the exposure of DEP and DIBP while for DEHP a major contribution is observed in low fat content foods for all age groups [9].

In general, considering the eight analyzed substances in this study, focused in fatty cereal based foods, low exposure data were obtained (0.00282–0.545 µg/kg body weight per day). However, it is important to consider that several contaminants were identified in the same pool sample (seven of the eight chemicals analyzed); consequently, the consumer are simultaneous exposed to multiple chemicals (cumulative exposure) through the diet from several sources (aggregate exposure) [53]. The known “cocktail effect” must be considered because the combination of a wide variety of chemicals could produce harmful effects in humans, even at low levels. In fact, in a draft update, the EFSA Panel on Food Contact Materials, Enzymes and Processing Aids (CEP Panel), based on a plausive common mode of action underlying the reproductive effects, propose a group-TDI for DEHP, DBP, DiNP and BBP and establish a value of 50 µg/kg body weight per day, expressed as DEHP equivalents [54].

## 4. Conclusions

An approach to estimate dietary exposure to certain chemicals transferred from plastic packaging materials into fatty cereal based foods is presented. Firstly, a GC–MS method was developed as a screening tool to identify migrants in the plastic packaging materials. More than 60 compounds of a different nature, such as plasticizers, antioxidants, slip agents, UV filters or photoinitiators were detected. Only 20 of 65 identified compounds were included in the positive list of European Regulation 10/2011. Secondly, selected migrants, namely, five phthalates (DEP, DIBP, DBP, DEHP and DNOP), the plasticizer ATBC, the antioxidant BHT and the UV filter octocrylene were determined in pooled food samples. The analytical method showed good linearity (R2 ≥ 0.9930) and an excellent sensitivity (LODs ≤ 0.01 µg/mL). Finally, the exposure assessment was performed; the mean dietary exposures ranged from 0.00282 to 0.545 µg/kg body weight per day depending on the analyte, being the highest exposure found for DEP in the group corresponding to 1–2 years. However, estimated exposures in all cases were found to be lower than the established tolerable daily intakes, when available.

## Figures and Tables

**Table 1:** Chemical structures and physicochemical characteristics of the target compounds.

Chemical Structure	Compound	CAS Number	Molecular Formula	Molecular Weight	Melting Point (°C)	Boiling Point (°C)	Density (g/cm^3^)	Vapor Pressure (Torr)	Log P (o/w)	SML (mg/kg)
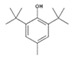	BHT	128-37-0	C_15_H_24_O	220.35	70 ^a^	265 ^a^	1.048 ^a^ (20 °C)	6.24E-3 ^b^	5.168 ^b^	3
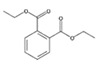	DEP	84-66-2	C_12_H_14_O_4_	222.24	−40.5 ^a^	298 ^a^	1.120 ^a^ (25 °C)	1.67E-3 ^b^	2.714 ^b^	NI
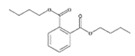	DBP	84-74-2	C_16_H_22_O_4_	278.34	−35 ^a^	340 ^a^	1.0465 ^a^ (20 °C)	1.08E-4 ^b^	4.752 ^b^	0.3 60 *
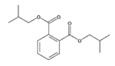	DIBP	84-69-5	C_16_H_22_O_4_	278.34	−64 ^a^	296.5 ^a^	1.039 ^a^ (20 °C)	1.54E-3 ^b^	4.440 ^b^	NI
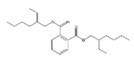	DEHP	117-81-7	C_24_H_38_O_4_	390.56	−55 ^a^	230 ^a^	0.9861 ^a^ (20 °C)	3.95E-6 ^b^	8.516 ^b^	1.5 60 *
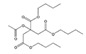	ATBC	77-90-7	C_20_H_34_O_8_	402.48	−80 ^a^	172–174 ^a^	1.046 ^a^ (25 °C)	3.35E-7 ^b^	5.227 ^b^	60 *
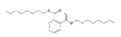	DNOP	117-84-0	C_24_H_38_O_4_	390.56	−25 ^a^	220 ^a^	0.978 ^a^ (20 °C)	3.84E-7 ^b^	8.828 ^b^	NI
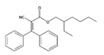	OCTO	6197-30-4	C_24_H_27_NO_2_	361.48	NA	478.5 ^b^	1.055 ^b^ (20 °C)	2.56E-9 ^b^	6.893 ^b^	0.05

^a^ Experimental information obtained from SciFinder ^®^ version web; ^b^ Predicted information obtained from SciFinder ^®^ version web; * Log P (octanol/water partition); Specific Migration Limit (SML) (T) (group restriction); NI: not included in the Regulation (EU) No 10/2011; NA: not available.

**Table 2:** Information of food packaging materials.

Coding	Sample Description	Type of Material		Thickness (µm)	Fat Content
		Internal Side	External Side		
AS_01	Snacks based in cereal	PP	PET	62	32.6 g/100 g (Saturated 9.1 g)
AS_02	Fried pork rinds	PP	PP	55	50 g/100 g (Saturated 16 g)
AS_03	Salty cookies	PP	PP	48	22 g/100 g (Saturated 19 g)
AS_04	Butter-flavored baked appetizer product	PP	PP	51	18.6 g/100 g (Saturated 2.4 g)
PL_01	Butter-flavored popcorn	PP	PP	47	26.3 g/100 g (Saturated 2.4 g)
GA_01	Toasted biscuit	PP	PP	26	10 g/100 g (Saturated 5 g)
GA_02	Milk chocolate covered wheat biscuit	PVDC	NC	38	24 g/100 g (Saturated 13 g)

NC: Nitrocellulose; PET: Polyethylene terephthalate; PP: Polypropylene; PVDC: Polyvinylidene chloride.

**Table 3:** Compounds identified in the studied plastic packaging materials.

Compound	IUPAC Name	Formula	CAS Number	RT (min)	Possible Uses	SI	RSI	TC	SML (mg/kg)	Samples													
										AS-01		AS-02		AS-03		AS-04		PL-01		GA-01		GA-02	
										ACN	HEX	ACN	HEX	ACN	HEX	ACN	HEX	ACN	HEX	ACN	HEX	ACN	HEX
Nonanal	Nonanal	C_9_H_18_O	124-19-6	9.97	Aldehyde	875	896	I	NI			✓	✓			✓	✓	✓	✓	✓	✓	✓	✓
Isophorone	3,5,5-trimethylcyclohex-2-en-1-one	C_9_H_14_O	78-59-1	10.34	Component of solvents for finishes and stoving lacquer	812	846	II	NI											✓			
* Caprolactam	Azepan-2-one	C_6_H_11_NO	105-60-2	12.50	Monomer	818	823	III	15													✓	✓
Nonanoic acid	Nonanoic acid	C_9_H_18_O_2_	112-05-0	12.66	Lacquers, plastics and as plasticizer	796	831	I	NI											✓	✓		
* Triacetin	2,3-diacetyloxypropyl acetate	C_9_H_14_O_6_	102-76-1	13.85	Plasticizer	913	913	I	NI	✓	✓	✓	✓			✓	✓						
* 2,6-Toluene diisocyanate	1,3-diisocyanato-2-methylbenzene	C_9_H_6_N_2_O_2_	91-08-7	13.98	Polyurethane resin	873	898	III	ND									✓	✓				
* 2,4-Toluene diisocyanate	2,4-diisocyanato-1-methylbenzene	C_9_H_6_N_2_O_2_	584-84-9	14.05	Polyurethane resin	838	845	III	ND					✓	✓			✓	✓				
Diphenyl ether	Phenoxybenzene	C_12_H_10_O	101-84-8	14.82	Manufacturing plastics and rubber, solvent	864	871	III	NI				✓	✓	✓		✓	✓	✓	✓	✓	✓	✓
* 2,6-Di-tert-butyl-1,4-benzoquinone	2,6-di-tert-butylcyclohexa-2,5-diene-1,4-dione	C_14_H_20_O_2_	719-22-2	15.64	Degradation product of antioxidants	865	867	II	NI	✓	✓	✓	✓	✓	✓	✓	✓	✓	✓	✓	✓	✓	✓
* Butylated Hydroxytoluene	2,6-ditert-butyl-4-methylphenol	C_15_H_24_O	128-37-0	16.20	Antioxidant; stabilizer in hot-melt adhesives and coatings	866	882	II	3			✓	✓			✓	✓						
* 2,4-di-tert-butylphenol	2,4-ditert-butylphenol)-	C_14_H_22_O	96-76-4	16.23	Degradation product of antioxidants	871	896	I	NI	✓	✓	✓	✓	✓	✓	✓	✓	✓	✓	✓	✓	✓	✓
2-Naphthol	Naphthalen-2-ol	C_10_H_8_O	135-19-3	16.55	Dyestuffs and pigments	806	852	III	NI													✓	
1,5,9-Trimethyl-1,5,9-cyclododecatriene	(1Z,5Z,9Z)-1,5,9-trimethylcyclododeca-1,5,9-triene	C_15_H_24_	21064-19-7	16.90		828	831	I	NI			✓	✓	✓	✓	✓	✓	✓	✓	✓	✓	✓	✓
* Diethyl Phthalate	Diethylbenzene-1,2-dicarboxylate	C_12_H_14_O_4_	84-66-2	17.33	Plasticizer	868	915	I	NI	✓	✓	✓	✓	✓	✓	✓	✓	✓	✓	✓	✓	✓	✓
Isophorone diisocyanate	5-isocyanato-1-(isocyanatomethyl)-1,3,3-trimethylcyclohexane	C_12_H_18_N_2_O_2_	4098-71-9	17.36	Polyurethane resin	904	920	III	ND	✓		✓											
* Hexadecane	Hexadecane	C_16_H_34_	544-76-3	17.47	Alkane	877	884	I	NI			✓	✓			✓	✓	✓	✓				
* Benzophenone	Diphenylmethanone	C_13_H_10_O	119-61-9	17.97	Photoinitiator for UV-curing of inks	724	828	III	0.6				✓	✓	✓			✓	✓			✓	✓
Tributyl phosphate	Tributyl phosphate	C_12_H_27_O_4_P	126-73-8	18.01	Plasticizer	855	893	III	NI			✓	✓					✓	✓	✓	✓		
2,6-Diisopropylnaphthalene	2,6-di(propan-2-yl) naphthalene	C_16_H_20_	24157-81-1	18.41	Solvent	823	846	III	NI			✓	✓			✓	✓	✓	✓	✓	✓		✓
Methyl tetradecanoate	Methyl tetradecanoate	C_15_H_30_O_2_	124-10-7	19.04	Slip agent	807	840	I	NI	✓	✓	✓	✓	✓	✓	✓	✓	✓	✓	✓	✓	✓	✓
Myristic acid	Tetradecanoic acid	C_14_H_28_O_2_	544-63-8	19.45	Lubricant	860	866	I	+													✓	
3,5-Di-tert-butyl-4-hydroxybenzaldehyde	3,5-ditert-butyl-4-hydroxybenzaldehyde	C_15_H_22_O_2_	1620-98-0	19.49	Degradation product of antioxidant	833	857	II	NI	✓	✓	✓	✓	✓	✓	✓	✓	✓	✓	✓	✓	✓	✓
* Octadecane	Octadecane	C_18_H_38_	493-45-3	19.95	Alkane	844	883	I	NI			✓	✓	✓	✓	✓	✓	✓	✓				
2-ethylhexyl salicylate	2-ethylhexyl 2-hydroxybenzoate	C_15_H_22_O_3_	118-60-5	20.04	UV filter	871	878	I	NI					✓	✓					✓	✓		
3’,5’-Di-tert-butyl-4’-hydroxyacetophenone	1-(3,5-ditert-butyl-4-hydroxyphenyl) ethanone	C_16_H_24_O_2_	14035-33-7	20.07	Degradation product of antioxidant	700	771	II	NI								✓				✓		
Hexadecanal	Hexadecanal	C_16_H_32_O	629-80-1	20.16	Aldehyde	835	850	I	NI			✓	✓	✓	✓	✓	✓	✓	✓	✓	✓		
Isopropyl myristate	Propan-2-yl tetradecanoate	C_17_H_34_O_2_	110-27-0	20.22	Plasticizer for cellulosic, pigment dispersant and binder	763	780	I	NI			✓	✓										
Triethyl citrate	Triethyl-2-hydroxypropane-1,2,3-tricarboxylate	C_12_H_20_O_7_	77-93-0	20.37	Plasticizer and solvent for inks, adhesives, coatings	870	878	III	60											✓	✓		
* Diisobutyl phthalate	Bis(2-methylpropyl) benzene-1,2-dicarboxylate	C_16_H_22_O^4^	84-69-5	20.66	Plasticizer	857	883	I	NI	✓	✓	✓	✓	✓	✓	✓	✓	✓	✓	✓	✓	✓	✓
2,6-Di-tert-butyl-4-nitrophenol	2,6-ditert-butyl-4-nitrophenol	C_14_H_21_NO_3_	728-40-5	21.06	Antioxidant	814	834	III	NI											✓		✓	
Acetyl triethyl citrate	Triethyl 2-acetyloxypropane-1,2,3-tricarboxylate	C_14_H_22_O_8_	77-89-4	21.10	Plasticizer	836	862	I	NI											✓	✓		
7,9 Di-Tert-Butyl-1-Oxaspiro (4,5)Deca-6-9-Diene-2,8-Dione	7,9-ditert-butyl-1-oxaspiro[4 .5]deca-6,9-diene-2,8-dione	C_17_H_24_O_3_	82304-66-3	21.19	By-product of antioxidant	901	940	III	NI	✓	✓	✓	✓	✓	✓	✓	✓	✓	✓	✓	✓	✓	✓
* Methyl palmitate	Methyl hexadecanoate	C_17_H_34_O_2_	112-39-0	21.38	Intermediate for detergents, emulsifiers stabilizers, resins, plasticizers	775	760	I	NI			✓	✓									✓	✓
Benzenepropanoic acid, 3,5-bis(1,1-dimethylethyl)-4-hydroxy-, methyl ester	Methyl 3-(3,5-ditert-butyl-4-hydroxyphenyl)propanoate	C_18_H_28_O_3_	6386-38-5	21.44	Degradation product of antioxidant	787	809	II	NI			✓	✓			✓	✓	✓	✓	✓	✓	✓	✓
* Dibutyl phthalate	Dibutylbenzene-1,2-dicarboxylate	C_16_H_22_O_4_	84-74-2	21.74	Plasticizer	809	879	I	0.3	✓	✓	✓	✓	✓	✓	✓	✓	✓	✓	✓	✓	✓	✓
Palmitic acid	Hexadecanoic acid	C_16_H_32_O_2_	57-10-3	21.79	Varnish, slip agent degradant	899	910	I	+	✓								✓				✓	
Tetradecanamide	Tetradecanamide	C_14_H_29_NO	638-58-4	21.88	Adhesive	798	814	III	NI													✓	✓
* Eicosane	Eicosane	C_20_H_42_	112-95-8	22.20	Alkane	871	903	I	NI			✓	✓			✓	✓	✓	✓				
Isopropyl palmitate	Propan-2-yl hexadecanoate	C_19_H_38_O_2_	142-91-6	22.43	Lubricants, waxes	829	831	I	NI					✓	✓							✓	✓
Octadecanal	Octadecanal	C_18_H_36_O	638-66-4	22.45	Aldehyde	851	890	I	NI			✓				✓		✓		✓			
Tributyl citrate	Tributyl-2-hydroxypropane-1,2,3-tricarboxylate	C_18_H_32_O_7_	77-94-1	23.22	Plasticizer	825	848	III	NI											✓	✓		
* Heneicosane	Heneicosane	H_21_H_44_	629-94-7	23.25	Alkane	751	877	I	NI			✓	✓					✓	✓				
* Methylenediphenyl 4,4’-diisocyanate	1-isocyanato-4-[(4-isocyanatophenyl)methyl]benzene	C_15_H_10_N_2_O_2_	101-68-8	23.34	Polyurethane resin	861	884	III	ND	✓	✓	✓	✓	✓	✓		✓	✓	✓	✓	✓	✓	✓
Tributyl aconitate	Tributyl-(1E)-1-propene-1,2,3-tricarboxylate	C_18_H_30_O_6_	7568-58-3	23.81	Plasticizer	919	949	I	NI	✓	✓	✓	✓	✓	✓	✓	✓	✓	✓	✓	✓	✓	✓
Octadecanoic acid	Octadecanoic acid	C_18_H_36_O_2_	57-11-4	23.89	Lubricant	815	830	I	+	✓													
Dibutyl Sebacate	Dibutyldecanedioate	C_18_H_34_O_4_	109-43-3	23.94	Plasticizer	887	913	I	60	✓	✓												
* Hexadecanamide	Hexadecanamide	C_16_H_33_NO	629-54-9	24.08	Slip agent	791	818	III	NI			✓	✓			✓						✓	✓
* Acetyl tributyl citrate	Tributyl-2-acetyloxypropane-1,2,3-tricarboxylate	C_20_H_34_O_8_	77-90-7	24.75	Plasticizer	900	943	I	60	✓	✓	✓	✓	✓	✓	✓	✓	✓	✓	✓	✓	✓	✓
* Tricosane	Tricosane	C_23_H_48_	638-67-5	25.21	Alkane	862	885	I	NI	✓	✓	✓	✓	✓	✓	✓	✓	✓	✓	✓	✓	✓	✓
Octyl methoxy cinnamate	2-ethylhexyl-3-(4-methoxyphenyl)acrylate	C_18_H_26_O_3_	5466-77-3	25.43	UV filter agent	873	893	I	NI					✓	✓								
Dehydroabietic acid, methyl ester	Methyl-(1R,4aS,10aR)-1,4a-dimethyl-7-propan-2-yl-2,3,4,9,10,10a-hexahydrophenanthrene-1-carboxylate	C_21_H_30_O_2_	1235-74-1	25.67	Component of varnishes, printing inks and adhesives	830	868	II	NI	✓	✓	✓	✓			✓	✓						
Octadecanamide	Octadecanamide	C_18_H_37_NO	124-26-5	26.04	Slip agent	833	824	III	+			✓	✓			✓	✓					✓	✓
* Tetracosane	Tetracosane	C_24_H_50_	646-31-1	26.14	Alkane	823	860	I	NI	✓	✓	✓	✓	✓	✓	✓	✓	✓	✓	✓	✓	✓	✓
Dehydroabietic acid	(1R,4aS,10aR)-1,4a-dimethyl-7-propan-2-yl-2,3,4,9,10,10a-hexahydrophenanthrene-1-carboxylic acid	C_20_H_28_O_2_	1740-19-8	26.60	Solvent for printing inks	846	916	II	NI	✓								✓		✓		✓	
Diethylene glycol dibenzoate	2-(2-benzoyloxyethoxy)ethyl benzoate	C_18_H_18_O_5_	120-55-8	26.88	Plasticizer	851	915	I	NI			✓										✓	
* Antioxidant 425	2-tert-butyl-6-[(3-tert-butyl-5-ethyl-2-hydroxyphenyl)methyl]-4-ethylphenol	C_25_H_36_O_2_	88-24-4	27.05	Antioxidant	802	855	III	1.5			✓	✓	✓	✓	✓	✓	✓	✓			✓	✓
2,4-Dicumylphenol	2,4-bis(2-phenylpropan-2-yl)phenol	C_24_H_26_O	2772-45-4	27.05	Degradation product of antioxidant	804	834	III	NI									✓	✓				
* Bis(2-ethylhexyl) phthalate	Bis(2-ethylhexyl) benzene-1,2-dicarboxylate	C_24_H_38_O_4_	117-81-7	27.32	Plasticizer	825	831	I	1.5	✓	✓	✓	✓	✓	✓	✓	✓	✓	✓	✓	✓	✓	✓
cis-11-Eicosenamide	(Z)-icos-11-enamide	C_20_H_39_NO	10436-08-5	27.65	Adhesive and component of coatings	807	821	III	+	✓	✓									✓	✓		
* Octocrylene	2-ethylhexyl 2-cyano-3,3-diphenylprop-2-enoate	C_24_H_27_NO_2_	6197-30-4	28.34	UV filter	852	903	III	0.05			✓	✓	✓	✓	✓	✓	✓	✓	✓	✓	✓	✓
2-Monostearin	1,3-dihydroxypropan-2-yl octadecanoate	C_21_H_42_O_4_	621-61-4	28.90	Lubricant	769	810	I	NI							✓	✓			✓	✓		
* Di-n-octyl phthalate	Dioctyl benzene-1,2-dicarboxylate	C_24_H_38_O_4_	117-84-0	28.96	Plasticizer	800	860	I	NI	✓	✓												
* 13-Docosenamide	(Z)-docos-13-enamide	C_22_H_43_NO	112-84-5	29.40	Slip agent	872	890	III	+	✓	✓	✓	✓	✓	✓	✓	✓	✓	✓	✓	✓	✓	✓
* Squalene	2,6,10,15,19,23-hexamethyltetracosa-2,6,10,14,18,22-hexaene	C_30_H_50_	111-02-4	29.63	Plasticizer	916	921	I	NI	✓	✓	✓	✓	✓	✓	✓	✓	✓	✓	✓	✓	✓	✓
* Glycerol trioctanoate	2,3-di(octanoyloxy)propyl octanoate	C_27_H_50_O_6_	538-23-8	30.57	Lubricant	788	857	I	NI	✓	✓							✓	✓	✓	✓	✓	✓

* Confirmed using standard; TC: Cramer Toxicity; ND: not detected; NL: not included in the Regulation (EU) No 10/2011; +: Specific migration limit of 60 mg/kg.

**Table 4:** Linearity of target compounds and their, limit of detection (LOD) and limit of quantification (LOQ).

Compound	Equation	R^2^	LOD (µg/mL)	LOQ (µg/mL)	Range (µg/mL)	Quantification Ion	Qualifier Ion
BHT	y = 1.3579x − 0.0011	0.9999	0.001	0.0025	0.0025–2	205	220
DEP	y = 1.8091x − 0.0249	0.9994	0.005	0.01	0.01–2	149	177
DIBP	y = 2.5364x − 0.0151	0.9990	0.0025	0.005	0.005–2	149	150
DBP	y = 2.6487x − 0.0284	0.9990	0.001	0.0025	0.0025–2	149	150
ATBC	y = 0.1684x − 0.0108	0.9930	0.01	0.025	0.025–2	185	129
DEHP	y = 1.0437x − 0.0158	0.9960	0.01	0.025	0.025–2	149	167
Octocrylene	y = 0.1509x − 0.0037	0.9970	0.005	0.01	0.01–2	204	232
DNOP	y = 1.5027x − 0.0615	0.9970	0.01	0.025	0.025–2	149	279

**Table 5:** Concentration of target migrants in composite food samples with the recovery data and estimated dietary exposure (mean, P95) in Spanish child and adolescent population.

Compound	Concentration (µg/g)			Dietary Exposure (µg/kg Body Weight per Day)						TDI (µg/kg Body Weight per Day)
				Mean			P95			
	1–2 Years	3–9 Years	10–17 Years	1–2 Years	3–9 Years	10–17 Years	1–2 Years	3–9 Years	10–17 Years	
DEP	0.329 (98%)	0.279 (96%)	0.205 (92%)	0.545	0.273	0.0761	2.80	1.19	0.397	500
DIBP	0.0516 (90%)	0.0344 (102%)	0.102 (82%)	0.0869	0.0333	0.0378	0.442	0.145	0.197	
DBP	0.0370 (100%)	0.0250 (103%)	0.0426 (98%)	0.0618	0.0245	0.0159	0.315	0.107	0.0831	10
ATBC	0.0393 (118%)	0.0554 (114%)	0.0527 (113%)	0.0652	0.0539	0.0196	0.332	0.235	0.102	
BHT	0.0148 (73%)	0.0476 (75%)	0.00762 (74%)	0.0251	0.0471	0.00282	0.128	0.205	0.0147	
Octocrylene	0.0257 (83%)	0.0164 (80%)	0.0319 (79%)	0.0435	0.0157	0.0119	0.221	0.0682	0.0618	
DEHP	0.0949 (72%)	0.101 (90%)	0.524 (93%)	0.159	0.0995	0.194	0.808	0.433	1.01	50
DNOP	<LOQ (84%)	<LOQ (85%)	<LOQ (74%)	0.0209	0.0123	0.00463	0.106	0.0533	0.0242	
